# Normal Platelet Integrin Function in Mice Lacking Hydrogen Peroxide-Induced Clone-5 (Hic-5)

**DOI:** 10.1371/journal.pone.0133429

**Published:** 2015-07-14

**Authors:** Michael Popp, Ina Thielmann, Bernhard Nieswandt, David Stegner

**Affiliations:** Department of Experimental Biomedicine–Vascular Medicine, University Hospital and Rudolf Virchow Center, University of Würzburg, Würzburg, Germany; National Cerebral and Cardiovascular Center, JAPAN

## Abstract

Integrin αIIbβ3 plays a central role in the adhesion and aggregation of platelets and thus is essential for hemostasis and thrombosis. Integrin activation requires the transmission of a signal from the small cytoplasmic tails of the α or β subunit to the large extracellular domains resulting in conformational changes of the extracellular domains to enable ligand binding. Hydrogen peroxide-inducible clone-5 (Hic-5), a member of the paxillin family, serves as a focal adhesion adaptor protein associated with αIIbβ3 at its cytoplasmic tails. Previous studies suggested Hic-5 as a novel regulator of integrin αIIbβ3 activation and platelet aggregation in mice. To assess this in more detail, we generated *Hic-5-null* mice and analyzed activation and aggregation of their platelets *in vitro* and *in vivo*. Surprisingly, lack of Hic-5 had no detectable effect on platelet integrin activation and function *in vitro* and *in vivo* under all tested conditions. These results indicate that Hic-5 is dispensable for integrin αIIbβ3 activation and consequently for arterial thrombosis and hemostasis in mice.

## Introduction

Vessel wall injury results in the exposure of the subendothelial extracellular matrix which initiates stable platelet adhesion and aggregation [1,2]. These processes are crucial for normal hemostasis, but in diseased vessels they may lead to pathological thrombus formation and infarction of vital organs [3]. Platelet adhesion and aggregation are mediated by integrins, heterodimeric transmembrane receptors composed of α and β subunits that are expressed in a low affinity state under resting conditions. The major platelet integrin αIIbβ3 is present in high copy numbers (up to 100,000 per platelet) and its function is well characterized. Integrin αIIbβ3 binds several ligands each containing an arginine-glycine-aspartic acid (RGD) sequence, such as fibrinogen, fibrin, vWF, fibronectin, thrombospondin and vitronectin [3]. After activation mediated by other platelet receptors, integrins shift to a high affinity state and efficiently bind their ligands [4]. This process requires the transmission of signals from the small cytoplasmic tails to the large extracellular domains of the integrin subunits [5]. Several proteins, including talin and kindlin-3, have been proposed to be involved in regulation of integrin activation [6–12]. A functional role for these proteins in integrin activation *in vivo* could not be confirmed in all cases as, for example, in the case of RIAM [13]. Recently, Kim-Kaneyama and colleagues reported that *hydrogen peroxide-induced clone-5* (Hic-5) acts as a novel regulatory factor for integrin αIIbβ3 activation and platelet aggregation in mice [14]. Hic-5 was identified as a gene inducible by transforming growth factor β1 (TGFβ1) as well as hydrogen peroxide in a differential screen of cDNA libraries from the mouse osteoblastic cell line MC3T3-E1. The authors of that study speculated that Hic-5 has a role in the growth-inhibitory pathway associated with *in vitro* senescence and that down-regulation of Hic-5 contributes to tumorigenesis [15]. Hic-5 contains four LIM domains at the C-terminus and shares high homology with paxillin that has been shown to localize to focal adhesions and to interact with talin in platelets [16,17]. In addition, it has been recently shown that paxillin negatively regulates platelet signaling pathways resulting in augmented αIIbβ3 activation upon stimulation of glycoprotein VI (GPVI) or G protein-coupled receptors (GPCR) [18].

We generated Hic-5-null (*Tgfb1i1* ^*-/-*^) mice and found, in stark contrast to the study by Kim-Kaneyama *et al*. [14], unaltered platelet integrin function *in vitro* and *in vivo*.

## Material and Methods

### Animals

Animal studies were approved by the district government of Lower Franconia (Bezirksregierung Unterfranken; AZ 54/11). *Tgfb1i1* ^*+/-*^ mice were generated by injection of embryonic stem cell clone EPD0817_1_D04 (KOMP) into blastocysts of pseudo-pregnant C57BL/6 females to generate germ line chimeras. Male chimeras were bred to C57BL/6 females to generate *Tgfb1i1* ^*+/–*^ mice, which were intercrossed to produce *Tgfb1i1* ^*–/–*^ mice and littermate wild-type controls. Mice were genotyped by PCR, with 5’ GGGACGGGGCGTAGATAAAG 3’ and 5’ GTGCAGCCCAGATTGTCTCT 3’ for the wild-type, and 5’ TAGATAGAGATGGCGCAACG 3’ and 5’ ACACCCATTCACACACTGGA 3’ for the *Hic-5-*null allele.

### Chemicals and antibodies

Midazolam (Roche Pharma AG), fentanyl (Janssen-Cilag GmbH) and the antagonists atipamezol (Pfizer), flumazenil and naloxone (both from Delta Select GmbH) were used according to the regulation of the local authorities. ADP, human fibrinogen, indomethacin and polyclonal anti-GAPDH antibody (Antibody ID: AB_796208) were from Sigma-Aldrich (Schnelldorf, Germany). Fibrillar type I collagen (Horm) was from Nycomed (Munich, Germany). Integrilin was from GlaxoSmithKline (Munich, Germany). U46619 was from Enzo Life Sciences GmbH (Lörrach, Germany), thrombin was from Roche (Mannheim, Germany), apyrase type III was from GE Healthcare (Chalfont St. Giles, UK), low-molecular-weight heparin was from ratiopharm GmbH (Ulm, Germany). Monoclonal anti-Hic5 antibody (Antibody ID: AB_398703) and monoclonal anti-CD29 antibody (Antibody ID: AB_393729) were from BD Biosciences (Heidelberg, Germany). Polyclonal anti-paxillin antibody (Antibody ID: AB_315576) was from Biolegend (Fell, Germany), polyclonal anti-leupaxin antibody (clone: RB37022) was from Biorbyt (Cambridge, United Kingdom). Polyclonal anti-vWF antibody (Antibody_ID: AB_2315604) and polyclonal rabbit anti-mouse IgG–horse-radish peroxidase (HRP) (P026002) were from Dako Cytomation (Hamburg, Germany). Anti-rabbit IgG-HRP (Antibody_ID: AB_2099233) was from Cell Signaling (Frankfurt, Germany). All monoclonal anti-platelet glycoprotein antibodies (host: rat), unconjugated or conjugated with fluorescein isothiocyanate (FITC), phycoerythrin (PE), DyLight 488 or HRP were obtained from EMFRET Analytics (Eibelstadt, Germany). Collagen-related peptide (CRP) was generated as previously described [19].

### RT-PCR analysis

Murine platelet mRNA was isolated with Trizol reagent, reverse transcribed and detected by RT-PCR, according to the manufacturer’s protocol (Invitrogen, Karlsruhe, Germany). The following primers were used to detect the *Hic-5* transcript: 5’ TTTTGGCCGCTGCCTTTAAC 3’ and 5’ AGGCTTGCATACTGTGCTGT 3’.

### Immunoblotting

Proteins of lysed platelets were separated by SDS-PAGE and blotted onto polyvinylidene difluoride membranes. After blocking, the membrane was incubated with antibody overnight at 4°C. HRP-conjugated antibodies were incubated for 1 h at room temperature, and enhanced chemilumincescence was used for visualization.

### Platelet preparation, aggregation and flow cytometry

Washed platelets were prepared as described previously [20]. For aggregometry, washed platelets (160 μL with 1.5x10^8^ platelets mL^-1^) were analyzed in the presence (CRP) or absence (thrombin) of 70 μg mL^-1^ human fibrinogen. Light transmission was recorded on a four-channel aggregometer (Fibrintimer; APACT, Hamburg, Germany) for 10 min and expressed in arbitrary units, with buffer representing 100% light transmission. For flow cytometry, heparinized whole blood was diluted 1:20 in Tyrode’s-HEPES buffer, incubated with saturating amounts of fluorophore-conjugated mAbs for 15 min at room temperature, and analyzed on a FACSCalibur (BD Biosciences, Heidelberg, Germany).

### Adhesion under flow conditions to collagen or vWF

For adhesion to collagen, cover slips were coated with 100 μg mL^-1^ collagen I at 37°C o/n. For adhesion to vWF, cover slips were coated with rabbit anti-human vWF antibody at 4°C o/n, washed with PBS, blocked for 1 h with 1% BSA in H_2_O and incubated with 100 μl murine serum obtained from control mice.

Blood (700 μl) was collected into 300 μl heparin (20 U/ml in TBS, pH 7.3). Whole blood was diluted 2:1 in Tyrode’s buffer containing Ca^2+^ and filled into a 1 ml syringe. For adhesion to collagen, before perfusion, anticoagulated blood was incubated with Dylight-488–conjugated anti- GPIX derivative (0.2 μg/mL) at 37°C for 5 minutes.

Transparent flow chambers with a slit depth of 50 μm, equipped with the coated coverslips, were connected to a syringe that was filled with diluted whole blood. Perfusion was performed using a pulse-free pump under high shear stress equivalent to a wall shear rate of 1000 s^-1^ (collagen I) or 1,700 s^-1^ (vWF).

Aggregate formation was visualized with a Zeiss Axiovert 200 inverted microscope (40x/0.60 objective). Phase-contrast and fluorescence pictures were recorded with a CoolSNAP-EZ camera, and analyzed off-line using MetaVue software.

Adhesion of platelets to bound vWF started 90 sec after perfusion of the slide. Thereafter, cover slipes were washed by a 4 min perfusion with Tyrode’s buffer at the same shear rate and phase-contrast images were recorded from at least five different microscopic fields (40x/0.60 objective). Image analysis was performed off-line using MetaVue software.

### Platelet spreading on fibrinogen

Cover slips were coated with 100 μg mL^-1^ human fibrinogen and blocked with 1% BSA in PBS. After rinsing with Tyrode-HEPES buffer, washed platelets (100 μL with 0.03x10^6^ platelets μL^-1^) were either activated with 0.01 U mL^-1^ thrombin or incubated with the inhibitors apyrase (ATP scavenger; 2U/ml) and indomethacin (inhibitor of COX-1/2; 1.4 μM) and allowed to spread on the cover slips. At the indicated time points, platelets were fixed with PBS 4% PFA and platelets were visualized with a Zeiss Axiovert 200 inverted microscope (100x/1.4 oil objective). Digital images were recorded using a CoolSNAP-EZ camera (Visitron) and analyzed off-line using MetaVue software.

### Platelet spreading on vWF

Glass coverslips were coated with a polyclonal rabbit anti-human vWF antibody at 4°C o/n, washed with PBS, blocked for 1 h with 1% BSA in H_2_O and incubated with 100 μl murine serum obtained from control mice. Washed platelets at 0.3x10^6^ platelets/μl were diluted 1:2.3 in Tyrode’s with Ca^2+^, incubated with integrilin (40 μg/ml) and botrocetin (2 μg/ml) and were allowed to adhere on the prepared cover slips. At the indicated time points, platelets were fixed with PBS 4% PFA and platelets were visualized with a Zeiss Axiovert 200 inverted microscope (100x/1.4 oil objective). Digital images were recorded using a CoolSNAP-EZ camera (Visitron) and analyzed off-line using MetaVue software.

### Clot retraction

Clot retraction studies were performed at 37°C in an aggregometer tube containing diluted PRP (platelet rich plasma) (3×10^5^ platelets μL^-1^), thrombin (5 U mL^-1^), and CaCl_2_ (20 mmol L^-1^). Clot retraction was recorded with a digital camera over a time span of 4.5 hours after activation.

### Intravital microscopy of thrombus formation in FeCl_3_-injured mesenteric arterioles

Four-week-old mice were anesthetized, and the mesentery was exteriorized through a midline abdominal incision [21]. Arterioles (35–60 μm diameter) were visualized with a Zeiss Axiovert 200 inverted microscope (10x /0.3 NA objective) (CarlZeiss, Göttingen, Germany) equipped with a 100-W HBO fluorescent lamp source, and a CoolSNAP-EZ camera (Visitron). Digital images were recorded and analyzed off-line with MetaVue software. Injury was induced by topical application of a 3 mm^2^ filter paper saturated with FeCl_3_ (20%). Adhesion and aggregation of fluorescently labeled platelets (DyLight-488-conjugated anti-GPIX IgG-derivative) in arterioles were monitored for 40 min or until complete occlusion occurred (blood flow stopped for > 1 min).

### Bleeding time

Mice were anesthetized, and a 1 mm segment of the tail tip was removed with a scalpel. Tail bleeding was monitored by gently absorbing blood with filter paper at 20 s intervals without making contact with the wound site. When no blood was observed on the paper, bleeding was determined to have ceased. Experiments were stopped after 20 min. For the saline model, 1 mm of the tail tip was amputated, and the tails were immersed in 0.9% isotonic saline at 37°C. The time to complete arrest of bleeding (no blood flow for 1 minute) was determined.

### Data analysis

The presented results are mean ± SD from three independent experiments per group, if not otherwise stated. Differences between control and Hic-5-null mice were statistically analyzed using the Mann-Whitney *U*-test. *P-*values < 0.05 were considered statistically significant.

## Results and Discussion

**Fig 1:**
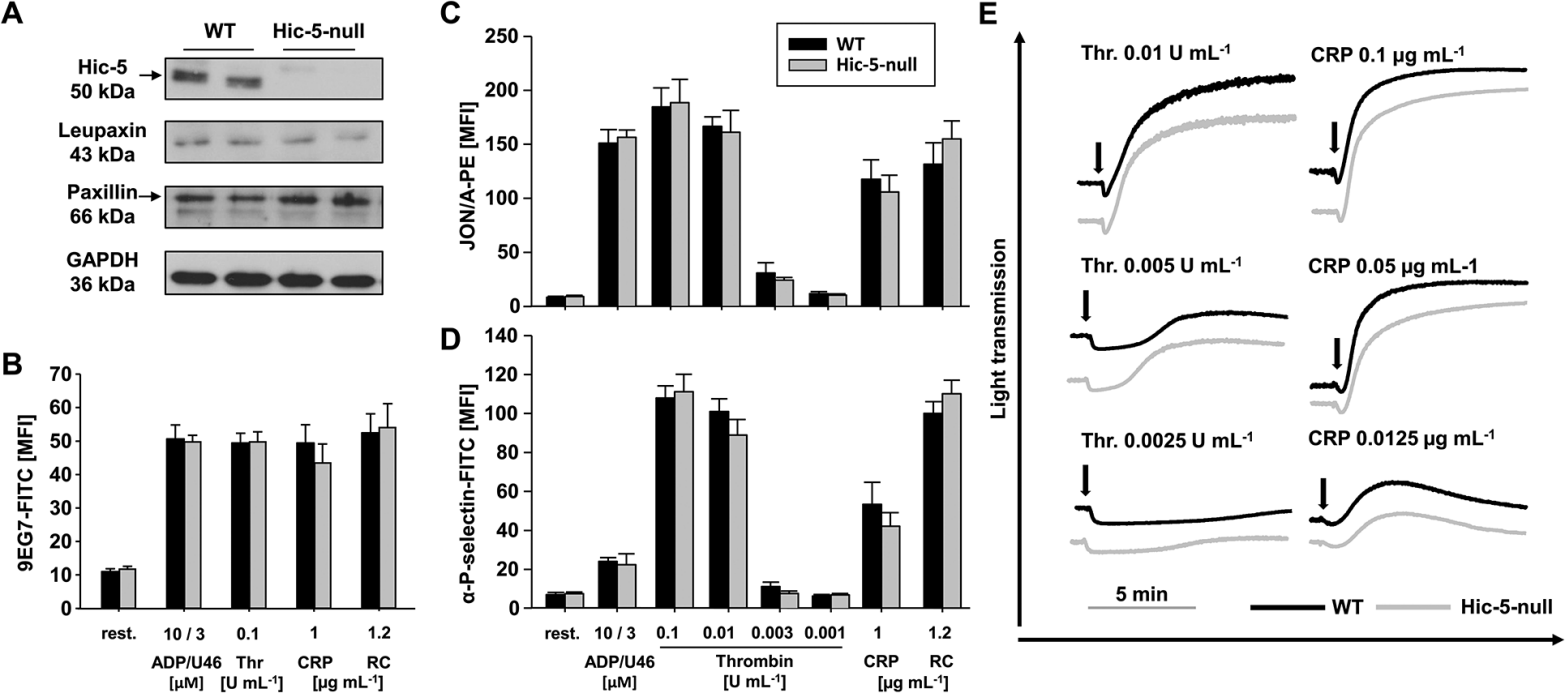
Hic-5 deficiency has no effects on inside-out activation of platelet integrins, degranulation and platelet aggregation. (A) Hic-5, leupaxin and paxillin expression was assessed by Western blot analysis. GAPDH served as loading control. (B) Flow cytometric analysis of β1 integrin activation (9EG7-FITC). (C, D) Flow cytometric analyses of (C) αIIbβ3 integrin activation (JON/A–phycoerythrin [PE]) and (D) degranulation-dependent P-selectin exposure in response to the indicated agonists. Results are mean fluorescence intensity (MFI) ± standard deviation (SD) of five mice per group. (E) Washed platelets were stimulated with the indicated agonists, and light transmission was recorded using a four channel aggregometer. Representative aggregation curves of three individual experiments are shown. CRP, collagen-related peptide, FITC, fluorescein isothiocyanate; Thr., Thrombin; RC, rhodocytin; U46, U46619; WT, wild-type.

We used embryonic stem cells (clone EPD0817_1_D04) provided by CSD, a collaborative team at the Children's Hospital Oakland Research Institute, and the University of California at Davis, within the Knockout Mouse Program (KOMP) with a knockout first approach targeting exon 1 of the *Tgfb1i1* gene leading to efficient loss of Hic-5 protein in *Tgfb1i1* ^*+/-*^ mice. *Tgfb1i1* ^*+/-*^ mice were intercrossed to obtain *Tgfb1i1* ^*-/-*^ mice (further referred to as Hic-5-null mice) and the respective control mice. Hic-5-null mice were born at normal Mendelian ratio, were viable and fertile and appeared overall healthy. The absence of Hic-5 in platelets was confirmed by Western blot analysis (Fig 1A) and RT-PCR (S1 Fig). The expression levels of the other paxillin family members leupaxin and paxillin were unaltered in Hic-5-null platelets compared to wild-type platelets (Fig 1A). The loss of Hic-5 did not alter platelet count or size and also red and white blood cell counts were indistinguishable from controls (Table 1). Flow cytometric measurements of major platelet surface receptors did not show significant alterations in receptor expression levels (Table 2) in Hic-5-null platelets compared to wild-type platelets. The unaltered basic blood and platelet parameters in the mutant mice indicate that Hic-5 plays no role for hematopoiesis and platelet morphology, in line with previous results of Kim-Kaneyama *et al*. [14].

**Table 1:** Basic blood and platelet parameters. Platelet count and size, as well as white blood cell count and red blood cell count were analyzed using a blood cell counter. *P* ≥ 0.05, n.s. WT, wild-type.

	WT	Hic-5-null	P
**Platelets μL** ^**-1**^ **[x10** ^**6**^ **]**	0.94 ± 0.13	0.93 ± 0.14	n.s.
**Platelet volume [fL]**	5.48 ± 0.15	5.63 ± 0.05	n.s.
**White blood cells μL** ^**-1**^ **[x10** ^**3**^ **]**	11.52 ± 2.44	11.20 ± 1.26	n.s.
**Red blood cells μL** ^**-1**^ **[x10** ^**6**^ **]**	7.23 ± 0.12	7.52 ± 0.48	n.s.

**Table 2:** Surface expression levels of platelet glycoproteins in wild-type and Hic-5-null mice. Expression of glycoproteins on the platelet surface was determined by flow cytometry. Diluted whole blood from the indicated mice was incubated with FITC-labeled antibodies at saturating concentrations for 15 minutes at room temperature, and platelets were analyzed directly. Data are expressed as mean fluorescence intensity ± SD. *P* ≥ 0.05, n.s. WT, wild-type; CLEC-2, C-type lectin-like receptor 2.

	WT	Hic-5-null	P
**GPV**	215 ± 6	218 ± 3	n.s.
**GPIb**	184 ± 4	192 ± 6	n.s.
**GPIX**	351 ± 16	354 ± 8	n.s.
**GPVI**	38 ± 2	37 ± 1	n.s.
**αIIbβ3**	420 ± 23	434 ± 17	n.s.
**α2**	44 ± 2	47 ± 7	n.s.
**β1**	117 ± 4	118 ± 3	n.s.
**CD9**	810 ± 14	781 ± 42	n.s.
**CLEC-2**	105 ± 3	106 ± 4	n.s.

**Fig 2:**
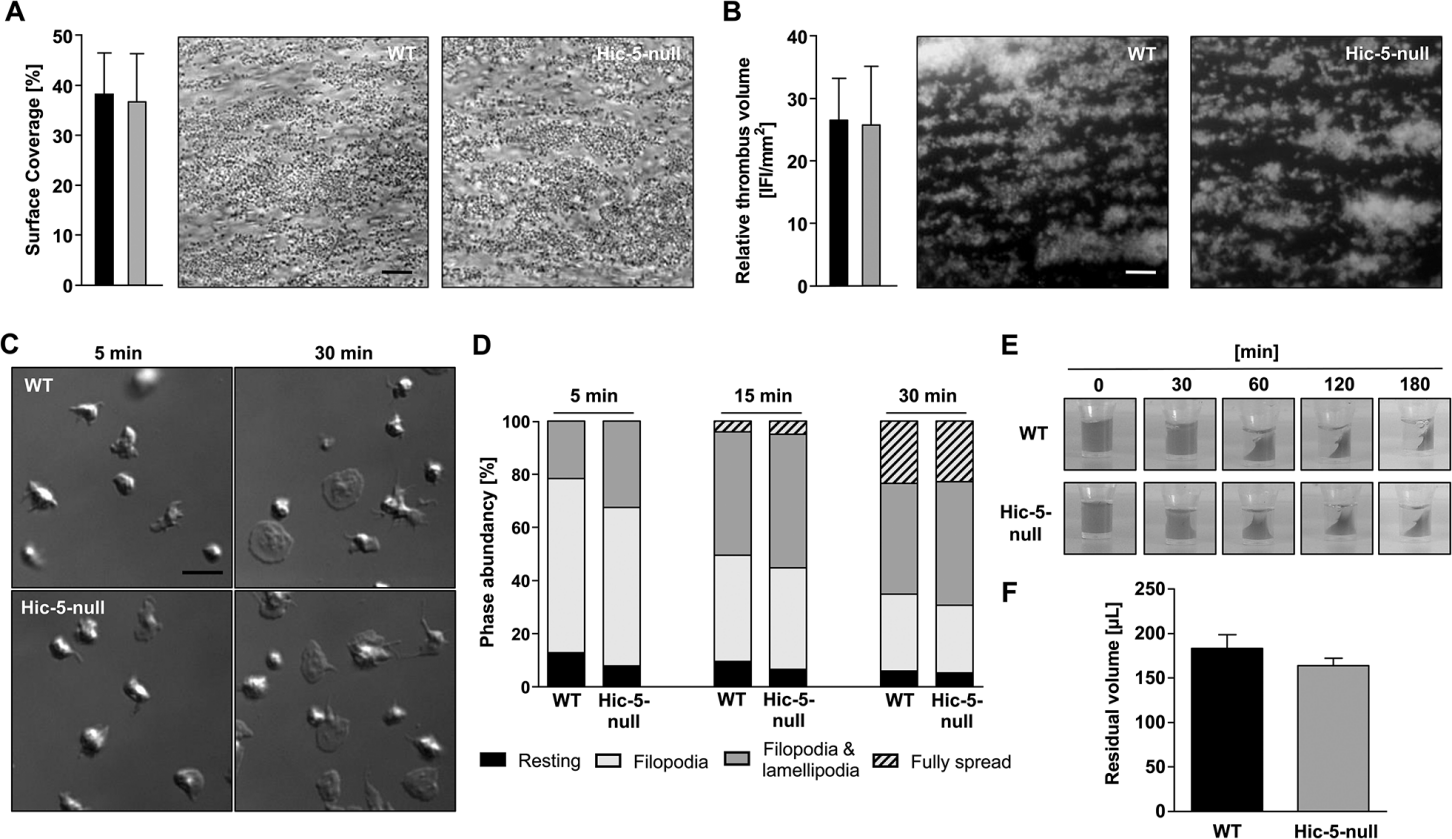
Lack of Hic-5 has no influence on platelet adhesion to collagen, spreading on fibrinogen and platelet integrin outside-in signaling *in vitro*. (A, B) Adhesion (A) and thrombus formation (B) of platelets on collagen was assessed in a flow adhesion assay at a wall shear rate of 1000 s^-1^. Scale bar, 25 μm. (C, D) Washed platelets of WT and Hic-5-null mice were allowed to spread on fibrinogen for up to 30 minutes after stimulation with 0.01 U mL^-1^ thrombin. (C) Representative images and (D) statistical evaluation of the percentage of spread platelets at different spreading stages are shown. Scale bar, 5 μm. (E, F) Clot formation in platelet-rich plasma was induced by the addition of thrombin (5 U mL^-1^) and 20 mmol L^-1^ CaCl_2_ and clot retraction was monitored over time (E). (F) Residual volume of serum after clot retraction was measured.

To clarify the role of Hic-5 deficiency on integrin activation and degranulation, activation of the major platelet integrin αIIbβ3 and surface exposure of α-granular P-selectin in response to different agonists was analyzed by flow cytometry. Stimulation of G-protein coupled receptors with ADP, U46619, a combination of both, or thrombin resulted in comparable results for wild-type and Hic-5-null platelets (Fig 1C and 1D). Similarly, Hic-5-null platelets reacted normally upon activation of the GPVI-ITAM (immunoreceptor tyrosine-based activation motif) pathway by CRP and in response to stimulation of the hemITAM receptor CLEC-2 (C-type lectin-like receptor 2) by rhodocytin (Fig 1C and 1D). β1-integrin activation was assessed by flow cytometry using the 9EG7-antibody, which selectively binds to the activated conformation of β1-integrins [22], and found to be indistinguishable between Hic-5-null and wild-type platelets (Fig 1B). In addition, standard aggregometry was performed, but no effects of Hic-5-deficiency on the aggregation response could be observed upon stimulation with any of the tested agonists (Fig 1E). In line with the previous data, Hic-5-null platelets showed normal adhesion (Fig 2A) and aggregate formation (Fig 2B) on collagen in a flow adhesion assay. These processes are strongly dependent on functional GPVI and α2β1 integrins [6,8].

These results demonstrate that Hic-5 is dispensable for platelet integrin activation downstream of both G protein–coupled receptors and for (hem)ITAM receptors. Notably, the unaltered integrin αIIbβ3 activation stands in contrast to the results of Kim-Kaneyama *et al*. [14], who found impaired integrin αIIbβ3 activation and reduced aggregation of Hic-5-null platelets in response to thrombin. In line with our study, Kim-Kaneyama *et al*. observed unaltered platelet degranulation in Hic-5-deficient platelets [14].

Ligand-occupied integrin αIIbβ3 mediates outside-in signaling, leading to cytoskeletal reorganization and platelet spreading [23]. Kim-Kaneyama *et al*. reported reduced filopodia length in Hic-5-deficient platelets [14]. In our experimental settings, Hic-5-null and wild-type platelets were allowed to spread on a fibrinogen-coated surface in the presence or absence of low concentrations of thrombin and found to form filopodia and lamellipodia with similar kinetics, and after 30 minutes, the number of fully spread platelets was comparable between both groups (Fig 2C and 2D and S2 Fig). Moreover, we did not observe differences in filopodia length. To further assess integrin outside-in signaling, we analyzed clot retraction [24] and found no differences in the kinetics and extent of this response between wild-type and Hic-5-null mice (Fig 2E and 2F). Together, these data exclude a major role of Hic-5 for integrin αIIbβ3 outside-in signaling in mouse platelets.

**Fig 3:**
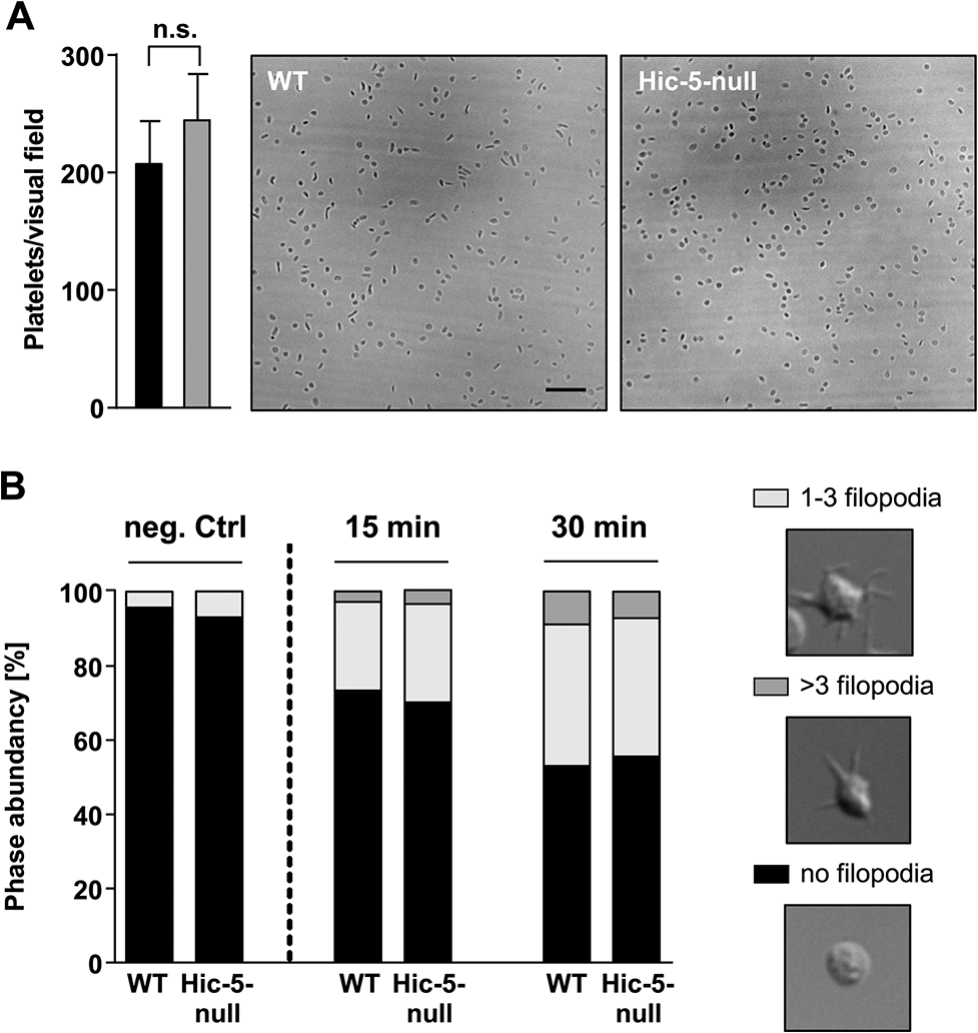
Hic-5-deficient platelets have unaltered GPIb function. (A) Whole blood of wild-type and Hic-5-null mice was perfused over a vWF-coated surface at a wall shear rate of 1,700 sec^-1^ and the number of adherent platelets was quantified. (B) Washed platelets were treated with integrilin (40 μg/ml) and botrocetin (2 μg/ml) and allowed to adhere to vWF-coated cover slips. Filopodia formation was quantified according to the number of extensions per platelet at indicated the time points.

At sites of vascular injury, vWF is exposed on the subendothelial extracellular matrix (ECM). The interaction between the platelet GPIb-V-IX receptor complex and vWF is important for platelet tethering, the initial step during the formation of a hemostatic plug. A potential role of Hic-5 in GPIb-mediated processes was assessed by monitoring the adhesion of platelets on immobilized vWF under high shear conditions. Hic-5-null and wild-type platelets attached to the immobilized vWF and in part firmly adhered to the surface to comparable extent and with similar kinetics (Fig 3A). In addition, we analyzed platelet spreading on a vWF-coated matrix under conditions of αIIbβ3 integrin-blockade. The stimulation of platelets by a GPIb specific signal results in a shape change, which is limited to contraction of the cell body and filopodia formation [25]. Both, Hic-5-null and wild-type platelets extended comparable numbers of filopodia (Fig 3B). Together, these results indicate that GPIb function is intact in Hic-5-deficient platelets.

**Fig 4:**
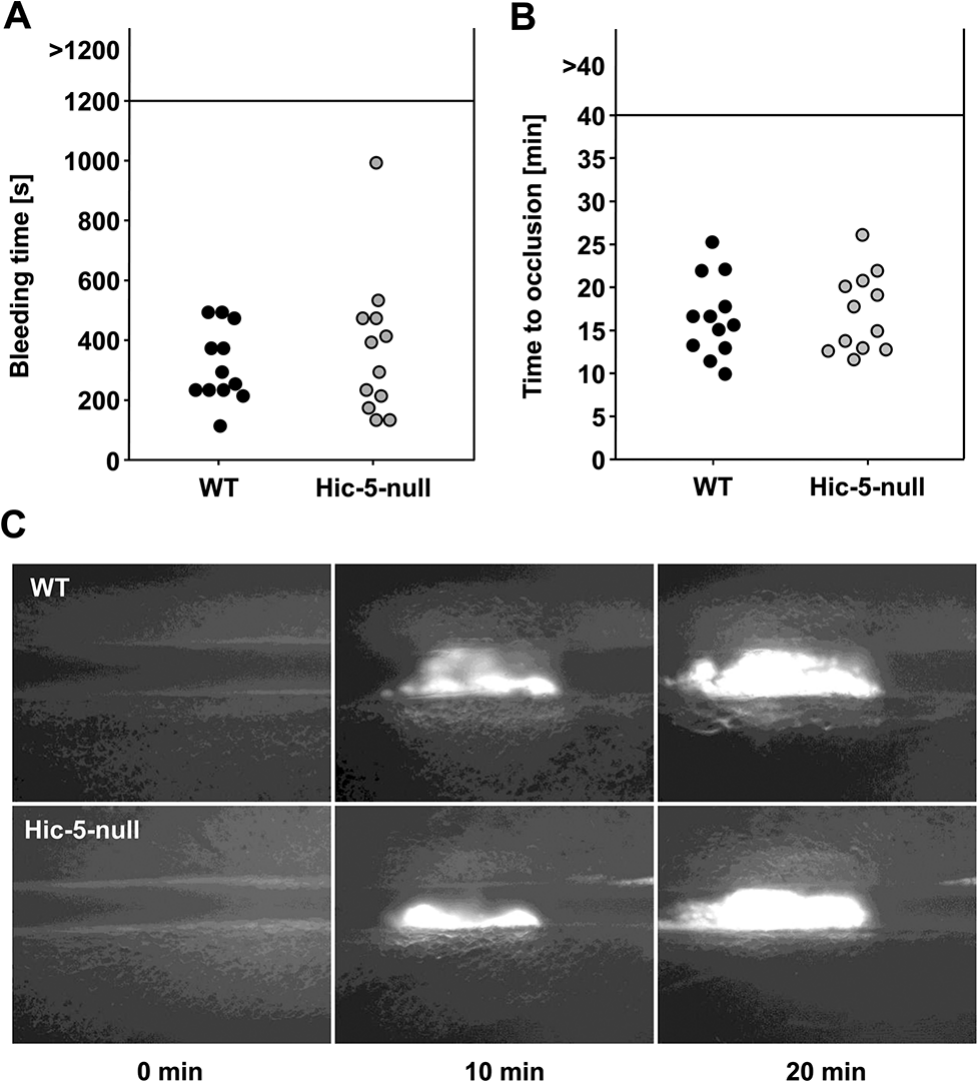
Unaltered *in vivo* thrombus formation in Hic-5-deficient mice. (A) Tail bleeding times of wild-type and Hic-5-null mice. Each symbol represents 1 animal. (B, C) Thrombus formation in small mesenteric arterioles was induced by topical application of 20% FeCl_3_. For monitoring of thrombus formation by intravital microscopy, platelets were labeled fluorescently. Time to stable occlusion (B) and representative pictures (C) of wild-type and Hic-5-null mice are shown. Each symbol represents one individual.

To investigate the role of Hic-5 in platelet function *in vivo*, we utilized two widely used tail bleeding time assays (filter paper and warm saline) and found comparable bleeding times for control and Hic-5-null mice (Fig 4A and S3 Fig respectively). This result stands in contrast to the study of Kim-Kaneyama *et al*. who found prolonged bleeding times in Hic-5-null mice using the saline method [14].

Kim-Kaneyama *et al*. reported that Hic-5-null mice are more resistant to experimental thromboembolism than control mice [14]. Consistent with our *in vitro* results, pathological thrombus formation, as assessed by intravital microscopy of FeCl_3_-injured mesenteric arterioles, was indistinguishable between wild-type and Hic-5-null mice, resulting in similar occlusion times for both groups (Fig 4B and 4C). These data indicate a dispensable role of Hic-5 in thrombus formation *in vivo*.

In this study, constitutive knockout mice for Hic-5 were used to assess the role of this focal adhesion protein in platelet function *in vitro* and *in vivo*. We did not detect any alterations in integrin inside-out or outside-in signaling in the absence of Hic-5, arguing against an essential role of the adaptor in these processes. Consequently, hemostasis and experimental thrombus formation were not affected by the lack of Hic-5. It is difficult to explain the discrepancy between our study and that of Kim-Kaneyama *et al*. [14], but differences in the genetic background might contribute, as previously proposed as a possible explanation for differences observed in the phenotype of GPVI-deficient mice [26]. However, based on our results we suppose that Hic-5 plays only a minor role in platelet integrin signaling *in vitro* and *in vivo*. Presumably, Hic-5 deficiency can be compensated by the other paxillin family members, paxillin and leupaxin, which are both expressed in murine platelets [27]. This is supported by a study from Rathore and colleagues [27]. They could show, that in aggregating human platelets, Hic-5 was tyrosine phosphorylated and recruited Csk (C-terminal Src kinase) via its SH2 domains to regulate SFK (Src family kinases) activity. However, in aggregating mouse platelets, Csk bound preferentilly to paxillin instead of Hic-5, although both are abundantly expressed.

It is possible that Hic-5 and the other paxillin family members share some tissue specific functions and that Hic-5 could play important roles in cells or tissues other than platelets. As an example, Hic-5 has been shown to have a role in the development of abdominal aortic aneurysms [28].

Notably, however, in contrast to mouse platelets human platelets express Hic-5 as the only paxillin family member [27]. Under these conditions, loss of Hic-5 could exert effects on human platelet function in thrombosis and hemostasis. To investigate this in mice, a mouse strain lacking all three paxillin members would be required.

## Supporting Information

S1 Fig Expression of *Tgfb1i1* mRNA. Analysis of the presence of *Tgfb1i1* mRNA in platelets by RT-PCR. GAPDH mRNA served as positive control. cDNA free sample was used as negative control.
(TIF)

S2 Fig Spreading on fibrinogen of unstimulated platelets. Washed platelets of WT and Hic-5-null mice were allowed to spread on fibrinogen for up to 60 minutes in the presence of apyrase (2 U/ml) and indomethacin (1.4 μM).
(TIF)

S3 Fig Normal hemostasis in Hic-5-null mice. Tail bleeding times in saline of wild-type and Hic-5-null mice. Each symbol represents 1 animal.
(TIF)
