# Supplementary figures and images for: Normal Platelet Integrin Function in Mice Lacking Hydrogen Peroxide-Induced Clone-5 (Hic-5)
Source: PLoS One. 2015 Jul 14;10(7):e0133429. doi: 10.1371/journal.pone.0133429 (PMC4501753; doi:10.1371/journal.pone.0133429)

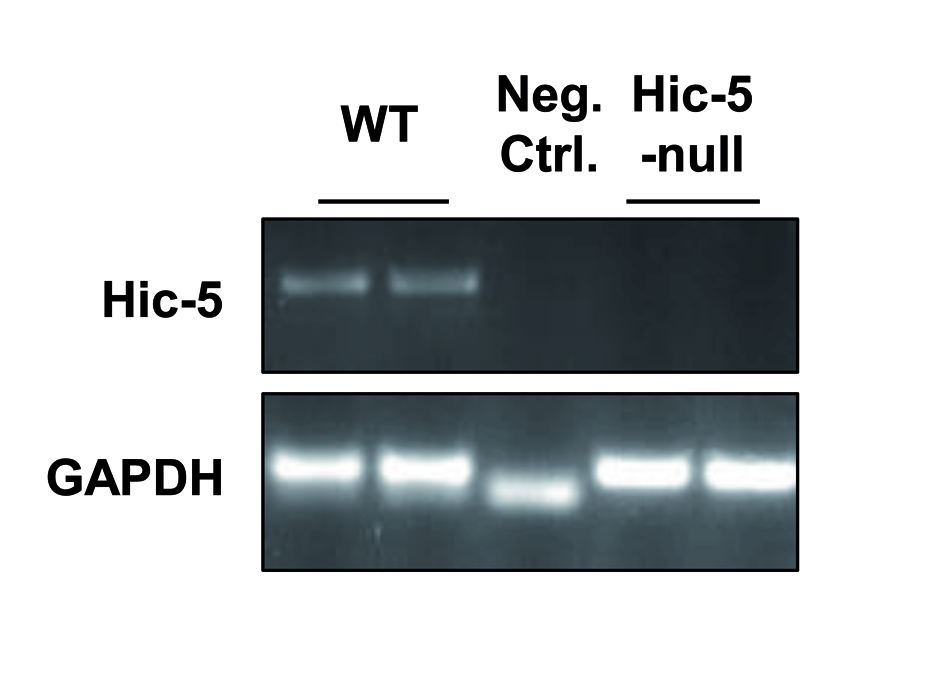

Supplement: S1 Fig — Analysis of the presence of Tgfb1i1 mRNA in platelets by RT-PCR. GAPDH mRNA served as positive control. cDNA free sample was used as negative control. (TIF) [file pone.0133429.s001.tif]

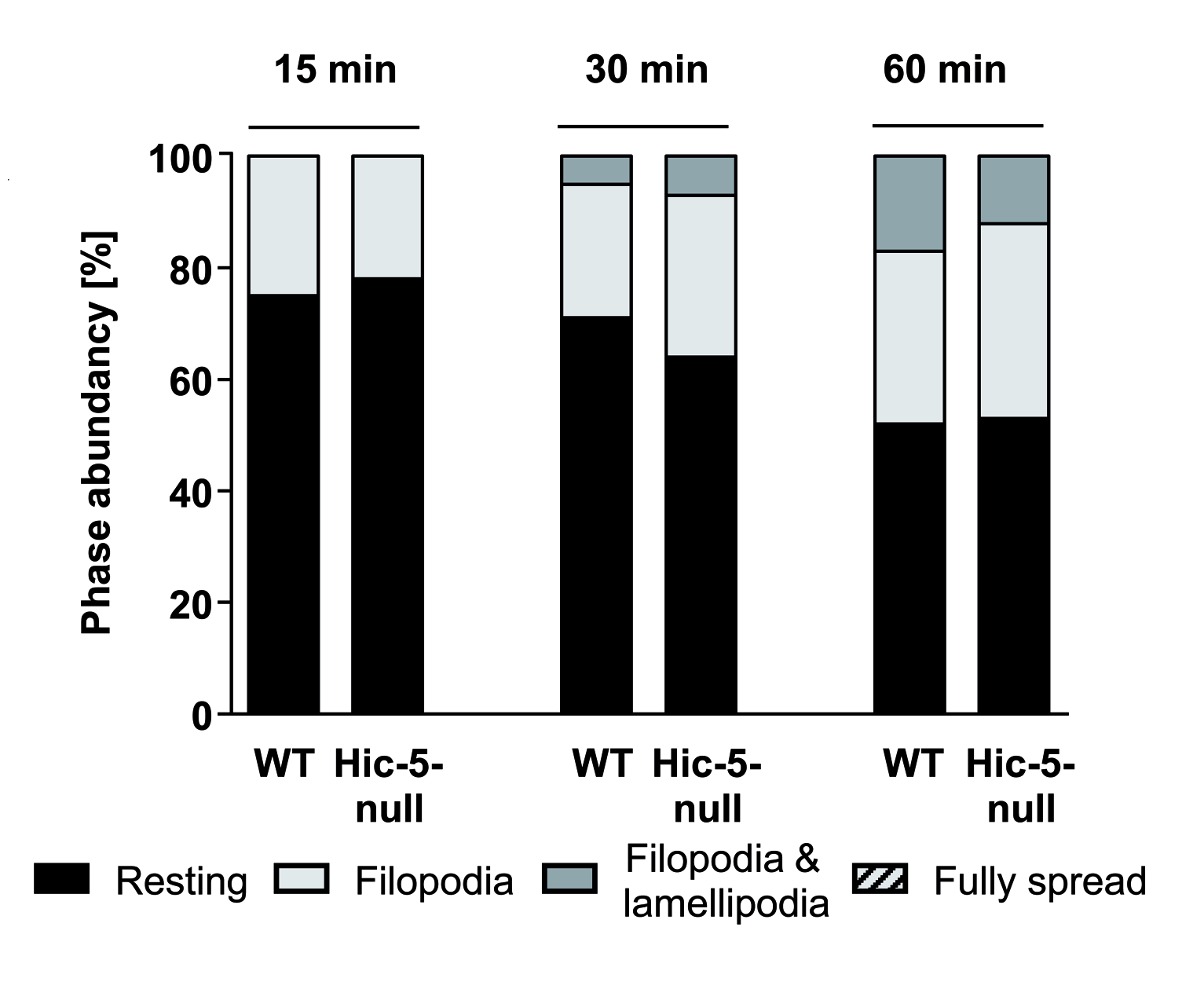

Supplement: S2 Fig — Washed platelets of WT and Hic-5-null mice were allowed to spread on fibrinogen for up to 60 minutes in the presence of apyrase (2 U/ml) and indomethacin (1.4 μM). (TIF) [file pone.0133429.s002.tif]

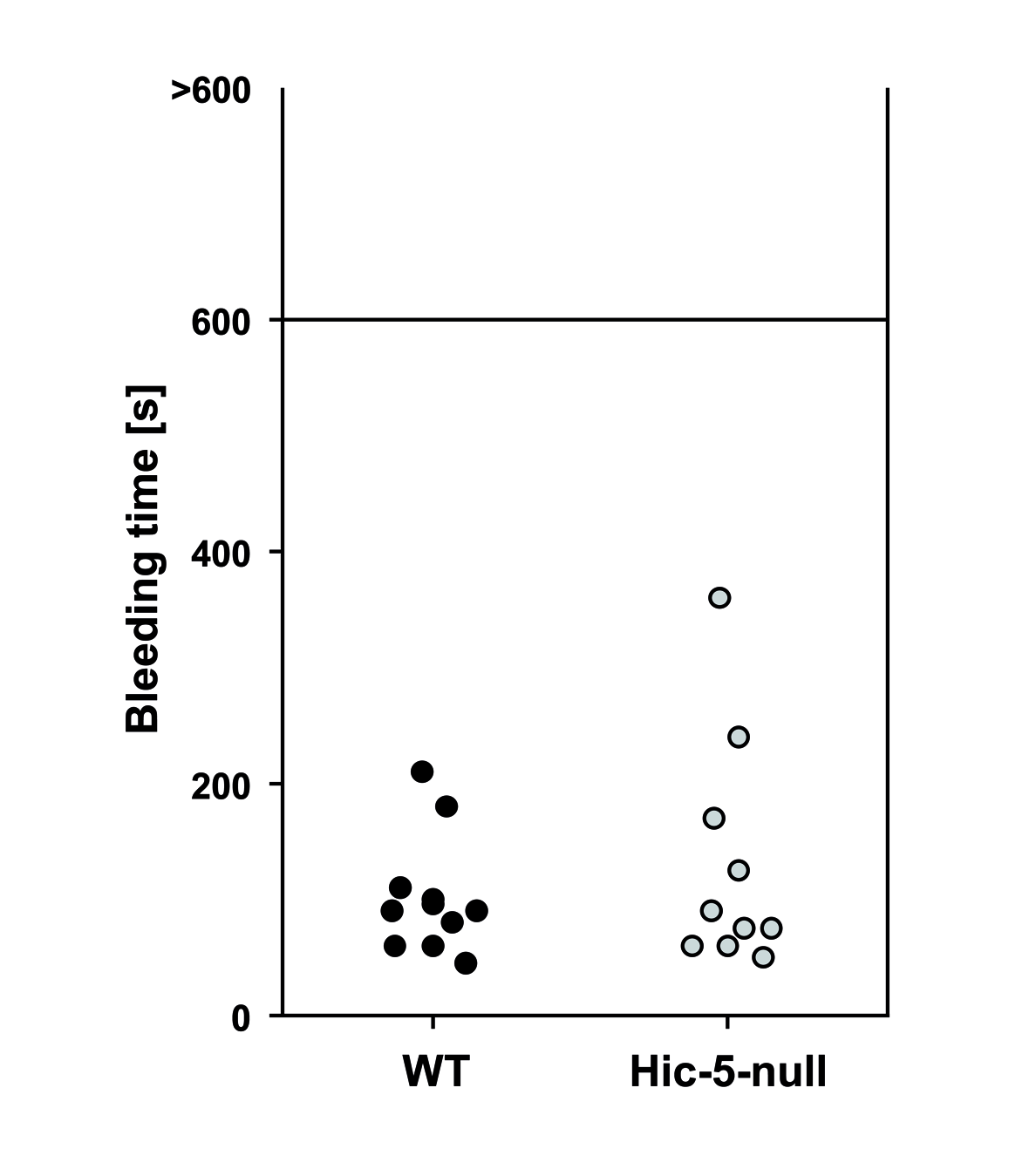

Supplement: S3 Fig — Tail bleeding times in saline of wild-type and Hic-5-null mice. Each symbol represents 1 animal. (TIF) [file pone.0133429.s003.tif]
